# Psychometric evaluation of the Work Readiness Questionnaire in schizophrenia

**DOI:** 10.1017/S1092852914000352

**Published:** 2014-10-01

**Authors:** Steven G. Potkin, Dragana Bugarski-Kirola, Chris J. Edgar, Sherif Soliman, Stephanie Le Scouiller, Jelena Kunovac, Eugenio Miguel Velasco, George M. Garibaldi

**Affiliations:** 1 Professor, Department of Psychiatry and Human Behavior, University of California–Irvine, Orange, California, USA; 2 Neuroscience Product Development (PDN), F. Hoffmann-La Roche Ltd, Basel, Switzerland; 3 Neuroscience Product Development (PDN), Roche Products Ltd., Welwyn Garden City, United Kingdom; 4 Product Development, Biometrics, Roche Products Ltd., Welwyn Garden City, United Kingdom; 5 Medical Director, Excell Research, Inc., Oceanside, California, USA; 6 Resolution Psychopharmacology Research Institute, Mendoza, Argentina

**Keywords:** clinical outcomes research, employment, function, readiness for work, schizophrenia

## Abstract

**Objective/Introduction:**

Unemployment can negatively impact quality of life among patients with schizophrenia. Employment status depends on ability, opportunity, education, and cultural influences. A clinician-rated scale of work readiness, independent of current work status, can be a valuable assessment tool. A series of studies were conducted to create and validate a Work Readiness Questionnaire (WoRQ) for clinicians to assess patient ability to engage in socially useful activity, independent of work availability.

**Methods:**

Content validity, test–retest and inter-rater reliability, and construct validity were evaluated in three separate studies.

**Results:**

Content validity was supported. Cronbach’s α was 0.91, in the excellent range. Clinicians endorsed WoRQ concepts, including treatment adherence, physical appearance, social competence, and symptom control. The final readiness decision showed good test–retest reliability and moderate inter-rater reliability. Work readiness was associated with higher function and lower levels of negative symptoms. Low positive and high negative predictive values confirmed the concept validity.

**Discussion:**

The WoRQ has suitable psychometric properties for use in a clinical trial for patients with a broad range of symptom severity. The scale may be applicable to assess therapeutic interventions. It is not intended to assess eligibility for supported work interventions.

**Conclusions:**

The WoRQ is suitable for use in schizophrenia clinical trials to assess patient work functional potential.

## Introduction

Unemployment among patients with schizophrenia is high.^1^ The Worldwide Schizophrenia Outpatient Health Outcomes (W-SOHO) study measured clinical and functional remission in 11,078 outpatients with schizophrenia from 37 countries. Unemployment rates were 55% in patients with functional remission and 88% in patients with neither functional nor clinical remission.^2^ In contrast, the global unemployment rate is 6.2%.^3^ Even for subpopulations with high unemployment, such as youths (15–24 years) in the Middle East and North Africa, the rate is only 25%.^3^

Unemployment affects patient quality of life due to insufficient daytime activities, lack of company and relationships, and few opportunities to increase self-esteem. Family and societal concerns focus on long-term financial impact and patients’ ability to live independently.^4^ A survey of patients, relatives, physicians, and payers showed payers ascribing the highest value to treatment goals affecting costs, including employment.^5^ Thus, ability to work is a legitimate schizophrenia treatment target. Attempts to increase employment among those with schizophrenia have included supported employment and a variety of vocational rehabilitation approaches.

One way to assess work ability is to evaluate employment status. However, employment status among patients with schizophrenia is hindered by work availability, and may be influenced by socioeconomic factors and cultural influences including disability rules and the stigma associated with mental illness. Previous work history and education also predict work status.^6^ A surrogate endpoint—the ability to work—rather than actual employment, may be a measure of success of an intervention independent of personal history, socioeconomics, or cultural factors.

Functional status is correlated with work status in patients with schizophrenia; eg, the University of California–San Diego Performance-based Skills Assessment (UPSA) scores are correlated with level of engagement in work, volunteering, and schooling.^7^ The brief UPSA (UPSA-B) also predicted work status.^8^ However, these measures focus on prerequisite activities for employment (eg, ability to use a telephone or public transport) and were not developed to assess clinical factors influencing the ability to get and keep a job (eg, the ability to relate to peers/supervisors), nor has research considered work exclusively as the capacity for independent, paid employment. While there is considerable value in supported employment and the recovery movement approaches, we focused on the capacity to independently perform activities that could merit pay. To our knowledge, no clinician-rated assessment of the ability to work exists for patients with serious mental illness. Our objective was to develop such a scale—the Work Readiness Questionnaire (WoRQ)—and evaluate its utility as a clinical trials tool.

## Methods

We set out to create and validate a work readiness questionnaire independent of work status that is easy to use and practical for clinicians to assess and rate patient ability to engage in socially useful activity that could merit pay. The concept was based on the “readiness for discharge” questionnaire^9^ and was guided by clinical experience. The questionnaire was validated for (a) content validity, (b) reliability, and (c) construct validity.

### Experimental methods

#### Study 1: Content validity

The WoRQ is composed of 7 items (Table 1) that capture a patient’s readiness to work based on capacity to initiate and maintain useful activity that could merit pay, leading to a final dichotomous work readiness judgment. The questionnaire is completed using progress notes, medical records, and input from mental health professionals, family members, or caregivers (Table 1). The 7 items are graded as follows: “strongly agree,” “agree,” “disagree,” or “strongly disagree,” based on a patient’s ability to conduct daily activities, interact with others, and adhere to treatment, and based on others’ perceptions of patient appearance, behavior, and impulse control. These 7 items are not totaled but are used to aid in reaching the dichotomous work readiness judgment.

**Table 1:** Work Readiness Questionnaire (WoRQ v4.0). Instructions: This instrument defines work as any useful activity that could merit pay, and does not include work that requires an unusual level of supervision or rehabilitation work. Activities of daily living can include using public transportation and meal preparation, in addition to basic self-care. The judgment on work readiness is independent of whether a job is available to the patient. The 7 items below are provided as a guide for answering the final question in the box. Please read each statement below and select a response based on all sources of information available. The final question is a global judgment and not the sum of the previous items.

		Strongly agree	Agree	Disagree	Strongly disagree
Item	Description	1	2	3	4
Item 1	The patient generally adheres to a treatment plan, including medication.				
Item 2	The patient is able to carry out activities of daily living.				
Item 3	The patient is able to consistently keep appointments and schedules with only minimal assistance.				
Item 4	The patient would have adequate impulse control when interacting with authority figures, peers or coworkers, and potential customers.				
Item 5	The patient’s behavior would not make others uncomfortable in a work situation.				
Item 6	The patient’s appearance would not make others uncomfortable in a work situation.				
Item 7	The patient’s current symptoms would not interfere with the ability to hold a job.				
	Based on your clinical judgment, is this patient ready for work?	Yes		No	

Content validity was established cross-culturally via qualitative analyses of interviews with 11 practicing clinicians to gather insights on schizophrenia and work readiness and obtain feedback on the preliminary WoRQ. Respondents included psychiatrists and psychiatric nurses from clinical sites in North America, Asia, Europe, and Latin America. Potential respondents were selected based on the following key criteria: schizophrenia experts working closely with patients, fluency in English, and willingness to participate in a 1-hour interview to discuss respondents’ experiences with schizophrenia patients. The literature on positive and negative symptoms, and work readiness research in schizophrenia was reviewed using MEDLINE, PROQOLID, and Mapi Values (Mapi Research Institute). A literature-based conceptual framework was developed to guide respondent telephone interviews. Data analyses included response coding and comparing/contrasting to the conceptual framework and original questionnaire. The questionnaire was subsequently revised and finalized for use in studies 2 and 3.

#### Study 2: Inter-rater and test-retest reliability

Ten practicing psychiatrists assessed 12 videotaped schizophrenia patients twice within 4 weeks. Patients were recruited from a single U.S. center by advertising within a network of colleagues and group homes. All were outpatients, not involved in a clinical trial, met diagnostic criteria for schizophrenia (*Diagnostic Statistical Manual of Mental Disorders*, Fourth Edition [DSM-IV]), and were heterogeneous for psychopathology, disability, gender, ethnicity/race, marital status, and employment status/background. Treatment records were required. Patients and caregivers gave informed consent and received compensation for participating. Questionnaire rating instructions were provided before Session I. Patients were rated in a non-proscribed order using an interactive DVD, including all 12 patient videos and supporting information. Raters reviewed each video; supporting interviews (caregivers, mental health professionals); and the patient’s personal, medical, and psychiatric history and current treatment. Raters completed the WoRQ. This process was repeated after 4 weeks, and raters did not have access to original ratings (Table 2).

**Table 2:** Test–retest reliability between session I (baseline) and session II (3–4 weeks later)

WoRQ items	Session I mean (SD)	Session II mean (SD)	ICC (1,1)^*a*^
1. The patient generally adheres to a treatment plan including medication.	1.98 (0.601)	1.97 (0.517)	0.42
2. The patient is able to carry out activities of daily living.	2.28 (0.769)	2.29 (0.679)	0.58
3. The patient is able to consistently keep appointments and schedules with only minimal assistance.	2.36 (0.828)	2.38 (0.711)	0.57
4. The patient would have adequate impulse control when interacting with authority figures, peers or coworkers, and potential customers.	2.43 (0.763)	2.36 (0.754)	0.60
5. The patient’s behavior would not make others uncomfortable in a work situation.	2.75 (0.759)	2.67 (0.737)	0.66
6. The patient’s appearance would not make others uncomfortable in a work situation.	2.53 (0.798)	2.58 (0.752)	0.64
7. The patient’s current symptoms would not interfere with the ability to hold a job.	3.11 (0.742)	3.22 (0.597)	0.45

ICC=intra-class correlation coefficient; SD=standard deviation; WoRQ=Work Readiness Questionnaire.

a Intra-class correlation coefficient, where ICC (1,1)=(bms – wms)/(bms + (κ – 1) **·** wms), with κ=number of raters, bms=between-patient mean square, and wms=within-patient mean square from a one-way generalized linear model with patient included in the model as main effect.

#### Study 3: Construct validity

This was a global, cross-sectional, observational, stand-alone study. Adult patients with schizophrenia (n=200) received study information, gave consent, and had eligibility confirmed before a data collection visit. The patient and second respondent (family member/caregiver/clinical staff) were interviewed by a clinician who completed assessments. Raters were free to judge how to evaluate conflicting information from patients and second respondents in the work readiness evaluation. Of the recruited patients, 25% were working independently at the time of assessment. Data were collected for demographics, health/psychiatric history, work status, work readiness (WoRQ), function (Global Assessment of Functioning [GAF],^10^ Level of Functioning [LoF]^11^]), negative symptoms (Negative Symptom Assessment–4-item version [NSA-4]^12^), and symptom severity (Clinical Global Impression of Severity of Negative Symptoms [CGI-S-N]^13^]) (Table 2). All assessments were completed by the same rater, with the WoRQ assessed prior to the GAF, LoF, NSA-4, and CGI-S-N.

### Statistical methodology

#### Content validity

This was established as described above.

#### Test–retest reliability

Test–retest reliability was calculated using intra-class correlation coefficients (ICCs) (1,1) in Study 2 for the initial items from Sessions I and II for each rater, where ICCs were interpreted as 0–0.2, poor; 0.3–0.4, fair; 0.5–0.6, moderate; 0.7–0.8, strong; and > 0.8, excellent/near perfect reliability. For the dichotomous work readiness question, a tetrachoric correlation was performed to evaluate reproducibility between Sessions I and II.

#### Inter-rater reliability

Inter-rater reliability was assessed at each session using ICCs for items 1–7 (rated 1–4) in Study 2. Following Shrout and Fleiss’s notation,^14^ ^*^ score reproducibility among 10 raters for each item was tested using single-measure reliability ICC (2,1), where ICCs assess rating reliability by comparing the variability of different ratings of the same item to the total variation across all ratings and subjects. This ICC (2,1) application differs from the ICC (1,1) used for test–retest reliability. ICC (2,1) values were interpreted as per test–retest. For the dichotomous work readiness question, inter-rater reliability was assessed using percentage agreement and Fleiss’s κ for multiple raters (ranging from 0=chance to 1=complete agreement) and tetrachoric correlation for observed dichotomous or binary variables.

#### Construct validity

GAF score, LoF score, NSA-4 total score, and CGI-S-N rating were used to assess construct validity. Means were tested using *t* tests for the 2 levels of work readiness (yes/no). Cross-tabulations and Fisher exact tests were used to estimate the relationship between work readiness and categorical aspects of the measures. Spearman correlations between work readiness judgments and the criterion measures were calculated (excluding LoF score). Sensitivity, specificity, and positive and negative predictive value for actual work status were also evaluated.

#### Internal reliability

Internal consistency, or how the items relate to the underlying construct, of the WoRQ items was measured by Cronbach’s α.^15^ Values ≥0.7 are generally interpreted as indicating good reliability, and those ≥0.9 are indications of excellent reliability.^16^

#### Relationship between WoRQ items and overall rating

Regression analyses evaluated correlations between individual WoRQ items and overall WoRQ work readiness ratings.

## Results

### Study 1: Content validity

In-depth exploratory interviews with clinical experts guided preliminary questionnaire revisions and supported its content validity. Telephone interviews captured the perceived importance of patients’ (a) medication adherence, (b) insight, (c) desire to work, (d) physical appearance, (e) social competence, and (f) positive symptom control. Refinements to the preliminary WoRQ included separating behavior and appearance as 2 items, item resequencing for logic and coherence, and language changes to orient clinicians to the concept in each item, with examples illustrating practice patterns in respondents’ geographic areas.

### Study 2: Test–retest reliability

Test–retest reliability for the initial questions (1–7) was moderate: ICCs (1,1) ranged from 0.42–0.66 (Table 2). For the final work readiness judgment, test–retest reliability was good (tetrachoric correlation, 0.73) (Table 3).

**Table 3:** Test–retest reliability between session I (baseline) and session II (3–4 weeks later) on work readiness status

	Session I				Session II						
	Ready for work		Not ready for work		Ready for work		Not ready for work				
Dichotomous variable	n	(%)	n	(%)	n	(%)	n	(%)	% agreement	κ	Tetrachoric Correlation
Work readiness status	24	(20.0)	96	(80.0)	21	(17.5)	99	(82.5)	84.17	0.48034	0.7342

### Study 2: Inter-rater reliability

There was good agreement among raters in the assessment of work readiness (> 70% at first rating/Session I; >80% at second rating/Session II). Agreement by κ statistic and tetrachoric correlation was fair to moderate (0.32 and 0.53 at Session I and 0.43 and 0.69 at Session II, respectively). This apparent discrepancy in agreement between good percentage agreement and fair-to-moderate κ and ICC may reflect the different underlying assumptions of the statistical tests. The κ statistic estimates the level of agreement exceeding that expected by chance and assumes randomness in the ratings, or guessing. Since raters are not expected to guess at responses, this estimate may be conservative. The ICC (2,1) was calculated according to the notation of Shrout and Fleiss, as opposed to that more commonly reported in the literature, which uses an average measure accounting for less variability. Thus ICC (2,1) is a more conservative approach comparatively. Moderate agreement was seen among raters in their assessment of the 7 items preceding the work readiness judgment, with ICCs (2,1) of 0.23–0.54. Levels of agreement improved from Session I to Session II (Table 4).

**Table 4:** Inter-rater reliability of WoRQ items and work readiness

	Session I			Session II		
	n=10 raters and n=12 patients			n=10 raters and n=12 patients		
WoRQ items	ICC (2,1)^*a*^			ICC (2,1)^*a*^		
1. The patient generally adheres to a treatment plan including medication.	0.23			0.29		
2. The patient is able to carry out activities of daily living.	0.42			0.43		
3. The patient is able to consistently keep appointments and schedules with only minimal assistance.	0.37			0.40		
4. The patient would have adequate impulse control when interacting with authority figures, peers or coworkers, and potential customers.	0.43			0.40		
5. The patient’s behavior would not make others uncomfortable in a work situation.	0.47			0.52		
6. The patient’s appearance would not make others uncomfortable in a work situation.	0.40			0.54		
7. The patient’s current symptoms would not interfere with the ability to hold a job.	0.35			0.24		
Dichotomous variable	% agreement	κ	Tetrachoric correlation	% agreement	κ	Tetrachoric Correlation
Work readiness status	78.15	0.31713	0.53	83.52	0.42921	0.69

ICC=intra-class correlation coefficient; WoRQ=Work Readiness Questionnaire.

a Intra-class correlation coefficient, where ICC (2,1)=(bms – ems) / (bms + (κ – 1) ems) + (κ **·** (jms – ems/n)), with κ=number of raters, n=number of patients, bms=between-patient mean square, wms=within-patient mean square, jms=mean square for rater, and ems=error mean square from a two-way generalized linear model with patient and rater included in the model as main effects.

### Study 3: Construct validity

Patients were predominantly male (68.5%). Fewer males (38.7%) than females (55.6%) were rated ready to work. Age was similar in those rated ready to work (44.6, SD 12.03) and not ready to work (45.6, SD 12.07). With respect to educational background, the highest percentage of patients in both readiness groups had a high school diploma or equivalent (25.0% ready; 38.4% not ready). However, a greater percentage of those ready to work (14.8%) than not ready (4.5%) had a college degree (Table 5).

**Table 5:** Study 3: Patient demographics by work readiness status

		Work readiness status	
Parameter		Ready for work	Not ready for work
Gender	Male, n (%)	53 (60.2)	84 (75.0)
	Female, n (%)	35 (39.8)	28 (25.0)
Age	Mean (SD)	44.6 (12.03)	45.6 (12.07)
Work status	Independent work, n (%)	28 (31.8)	13 (11.6)
	Supported work, n (%)	5 (5.7)	4 (3.6)
	Unemployed, n (%)	55 (62.5)	95 (84.9)
Education	Some primary/elementary school	9 (10.2)	12 (10.7)
	Some high school	20 (22.7)	24 (21.4)
	Vocational school or certificate program	2 (2.3)	3 (2.7)
	High school diploma or equivalent	22 (25.0)	43 (38.4)
	Some college	20 (22.7)	23 (20.5)
	College or university degree (2- or 4-year)	13 (14.8)	5 (4.5)
	Graduate degree	2 (2.3)	2 (1.8)

SD=standard deviation.

Mean GAF scores were significantly higher (*p*<0.0001) in patients rated ready to work (61.8, SD 9.39) than those rated not ready (48.0, SD 10.34). Mean scores fell into the “some mild symptoms” and “serious symptoms” categories, respectively (Table 6).

**Table 6:** Mean difference in function and negative symptom severity by work readiness status

	Work readiness status Mean (SD)		
Variable	Ready for work	Not ready for work	*t* test p value
GAF score	61.8 (9.39)	48.0 (10.34)	< 0.0001
NSA-4 total score	11.6 (3.27)	14.7 (3.97)	< 0.0001
CGI-S-N score	3.2 (0.80)	4.1 (0.89)	< 0.0001

CGI-S-N=Clinical Global Impression of Severity of Negative Symptoms; GAF=Global Assessment of Functioning; NSA-4=Negative Symptom Assessment, 4-item version; SD=standard deviation.

The LoF also showed an association between better function and work readiness (Table 6). There was a correlation of 0.62 between better function and work readiness, and a greater proportion of patients ready to work achieved higher LoF ratings. All patients but 1 rated ready to work were in the “moderate” (55.7%) and “slight” (43.2%) impairment categories. Patients rated not ready to work were categorized as “moderate” (38.4%), “severe” (42.0%), and “most severe” (13.4%).

The mean NSA-4 total score was significantly lower (*p*<0.0001) in patients rated ready to work (11.6, SD 3.28) versus not ready to work (14.7, SD 3.97), reflecting lesser severity of negative symptoms in ready to work patients (Table 6).

The CGI-S-N mean was significantly lower (*p*<0.0001) in patients rated ready to work (3.2, SD 0.8) versus not ready to work (4.1, SD 0.9), reflecting lesser severity of global negative symptoms in ready to work patients (Table 6).

Agreement between current work and work readiness status showed 70% sensitivity, 63% specificity, 32% positive predictive value (PPV), and 89% negative predictive value (NPV) (Table 7).

**Table 7:** Analysis of agreement between current work status and work readiness status (current work status “Yes” corresponds to patients working independently ONLY, and sheltered employment has been converted to current work status “No”)

Measure of accuracy	Estimate
Sensitivity^*a*^	0.70
Specificity^*b*^	0.63
PPV^*c*^	0.32
NPV^*d*^	0.89

NPV=negative predictive value; PPV=positive predictive value.

a Proportion of patients ready to work among those who are employed.

b Proportion of patients not ready to work among those who are unemployed.

c Proportion of employed patients among those who are ready to work.

d Proportion of unemployed patients among those who are not ready to work.

These diagnostic tests consider current work status as “gold standard,” and work readiness as “test outcome.”

Cronbach’s α showed good internal consistency for the initial 7 items (α=0.89). Inclusion of the final dichotomous work readiness question increased internal consistency slightly (α=0.91), while item deletion did not result in any marked change in α (range 0.87–0.9).

### Study 3: Relationship between WoRQ items and overall rating

Logistic regression showed that 2 items—impulse control (#4) and interference of current symptoms (#7)—were significantly associated with the final work readiness judgment, accounting for 60% of the variance. Backward selection, wherein we removed the least significant item, resulted in the retention of just these 2 items in the model as still significant at the 0.05 level (Table 8).

**Table 8:** Logistic regression analysis of WoRQ: logistic regression individual items versus work readiness status

		95% Confidence interval		
WoRQ item	Odds ratio	Lower	Upper	*p* value
1. The patient generally adheres to a treatment plan including medication.	0.944	0.233	3.825	0.9359
2. The patient is able to carry out activities of daily living.	0.893	0.248	3.219	0.8623
3. The patient is able to consistently keep appointments and schedules with only minimal assistance.	0.596	0.162	2.189	0.4354
4. The patient would have adequate impulse control when interacting with authority figures, peers or coworkers, and potential customers.	0.360	0.131	0.989	0.0475^*a*^
5. The patient’s behavior would not make others uncomfortable in a work situation.	0.940	0.304	2.904	0.9142
6. The patient’s appearance would not make others uncomfortable in a work situation.	0.571	0.216	1.509	0.2581
7. The patient’s current symptoms would not interfere with the ability to hold a job.	0.026	0.008	0.083	<0.0001^*a*^

WoRQ=Work Readiness Questionnaire.

^a^Intra-class correlation coefficients according to Shrout and Fleiss’s notation.

## Discussion

The WoRQ, as established in patients with broad symptom severity, has suitable psychometric properties for use in a clinical trial setting to assess patients’ potential to work.

Practicing clinicians worldwide endorsed the concepts in the WoRQ: treatment adherence, physical appearance, social competence, and symptom control. Specific items addressing patient’s insight and willingness to work (concepts also endorsed by the validity panel) were considered implicit in the WoRQ items. Indeed, logistic regression showed a significant association of impulse control (impulse control when interacting with authority figures, peers or coworkers, and potential customers) and interference of current symptoms items with the final readiness judgment, confirming their importance. The WoRQ may be conceptualized as an ordered evaluation moving from basic to more complex functions with a more direct relation to work and symptom severity. Thus, items later in the scale will be more closely related to the final decision but may overlap with earlier items. Early items may provide a foundation for late-item judgments.

The items were internally consistent. Reliability was adequate; the final readiness decision showed good test–retest and moderate inter-rater reliability. There was support for improved inter-rater reliability from Session I to Session II among the psychiatrists who rated videos, which suggests that scale familiarity may be important for reliable ratings.

Work readiness was associated with significantly higher levels of functioning: on the GAF, this difference reflected a mean GAF score in the “serious symptoms” category for patients who were rated not ready to work, versus “some mild symptoms” for patients who were rated ready to work, with a similar pattern evident for the LoF. Patients who were rated ready to work also showed a lower level of negative symptoms (NSA-4) and global negative symptom severity (CGI-S-N).

The performance-based UPSA has shown to be predictive of work status and associated with other employment-related measures. For employment status, the UPSA-B has demonstrated low PPV (36%) but strong NPV (87%) (sensitivity was 75.9% and specificity was 59.0%).^8^ Similarly, the WoRQ showed low PPV (32%) but strong NPV (89%). For the UPSA-B, this result was attributed to a greater ability to identify patients unable to work versus able to work. For both scales, an added consideration is the availability of work and the probability that some patients able to work are unable to obtain employment, leading to misclassification. A possible advantage of the WoRQ is the use of a binary (yes/no) evaluation not reliant on a cut score. Mausbach et al^8^ used receiver operating characteristic curves to identify the most sensitive cut scores in order to evaluate work status, but acknowledged sample homogeneity (high levels of education, low levels of psychosis, Ashkenazi Jewish ethnicity) as a possible flaw. Thus, findings may be population-dependent, and cut scores may not be broadly applicable. The WoRQ underscores the importance of social competence evidenced by collaborative ability and impulse control as a necessary prerequisite for work readiness, while the UPSA focuses on daily living activities to indicate functional capacity. Thus, the WoRQ may have broader application across different cultures and heterogeneous populations. Finally, the WoRQ is easy to use and requires an average completion time of < 5 minutes, which is shorter than the UPSA-B (10–15 minutes).

The WoRQ may not be equally applicable to school-age and retirement-age patients, given its focus on work defined as “any useful activity that could merit pay.” Furthermore, although Study 3 recruited patients in the U.S., Sweden, and Argentina, they were predominantly Caucasian or African American. Evaluation of the WoRQ in wider multinational and cross-cultural samples is important to its future development for clinical trial use. Additionally, question content was not strongly supported by regression analyses, despite qualitative endorsement, possibly reflecting the ordered structure of the questionnaire. Thus, there may be modifications to the preceding item content that may help in final clinical evaluation, including explicit use of such concepts as motivation/desire to work and insight into psychiatric status. The NSA-4 scores, which distinguished those ready to work from those not, contains 2 items assessing decreased social drive and interests. Cognitive function also was not considered, but has been associated with employment status^17^ and may affect work readiness. These findings were based on cognitive task performance, and clinical evaluation of cognition may not show the same association. Interpreting data relating work status to the WoRQ is complicated by work subcategories. Although the WoRQ focuses on readiness for “unsupported” paid work, factors such as full- versus part-time, skilled versus unskilled, and the level of autonomy for patients in work were not specifically considered. Such comparisons would require evaluation in a larger sample. Finally, it should be acknowledged that since the intent of the questionnaire is to employ it as a research tool to determine readiness for full-time employment, that merits pay, and does not require an unusual level of supervision, the work readiness judgment is not intended to evaluate eligibility for supported work. Desire to work may be key in selecting individuals who can benefit from supported employment and other vocational rehabilitation approaches.^18^

## Conclusion

In conclusion, the WoRQ is a valid tool for use in evaluating patients’ readiness to work or conduct a useful activity meriting pay. It is an ordered evaluation that includes social competence, collaborative ability, and impulse control. It has the potential to evaluate a key aspect of recovery/functional remission in clinical practice, independent of the availability of paid employment. The sensitivity of WoRQ to therapeutic effects requires investigation. Also, longer-term data are needed to understand the predictive value of WoRQ to finding a job or conducting a useful activity.

## Disclosures

This work was supported by F. Hoffmann-La Roche. Steven G. Potkin has received grant support, funding, honoraria, or has been a paid consultant to the following companies: Amgen, Bristol-Myers Squibb, Concert Pharmaceuticals, Eli Lilly, Forum Pharmaceuticals, Genentech, Janssen Pharmaceutical, Lundbeck, Merck, Novartis, Otsuka, Sunovion, Roche, Takeda Pharmaceuticals International, Takeda Global Research and Development, and Toyama Pharmaceuticals. Jelena Kunovac has received grant support, funding, honoraria, or has been a paid consultant to the following companies: Otsuka and Sunovion. Dragana Bugarski-Kirola, Sherif Soliman and George Garibaldi are full-time employees of F. Hoffmann-La Roche. Chris J. Edgar and Stephanie Le Scouiller are full time employees of Roche Products Ltd. Eugenio Miguel Velasco does not have anything to disclose.

## Footnotes

* Regarding the notation of Shrout and Fleiss, the first number refers to the type of analysis of variance (ANOVA: random versus mixed model) and the number of factors included in the ANOVA, and the second number refers to whether the reliability is for a single rating or for an average of ratings obtained from more than one rater. ICC (1,1) corresponds to a one-way ANOVA with patient included as a random factor, where each patient is rated by a set of raters that is randomly selected from a larger population. ICC (2,1) corresponds to a two-way ANOVA with patients and raters included as random factors in order to assess whether the ratings of one rater are apt to be the same as for another, for individual patients.
